# Exploitation of Herpesviral Transactivation Allows Quantitative Reporter Gene-Based Assessment of Virus Entry and Neutralization

**DOI:** 10.1371/journal.pone.0014532

**Published:** 2011-01-17

**Authors:** Henrike Reinhard, Vu Thuy Khanh Le, Mats Ohlin, Hartmut Hengel, Mirko Trilling

**Affiliations:** 1 Institute for Virology, Heinrich-Heine-University, Düsseldorf, Germany; 2 Department of Immunotechnology, Lund University, Lund, Sweden; McMaster University, Canada

## Abstract

Herpesviral entry is a highly elaborated process requiring many proteins to act in precise conjunction. Neutralizing antibodies interfere with this process to abrogate viral infection. Based on promoter transactivation of a reporter gene we established a novel method to quantify herpesvirus entry and neutralization by antibodies. Following infection with mouse and human cytomegalovirus and Herpes simplex virus 1 we observed promoter transactivation resulting in substantial luciferase expression (>1000-fold). No induction was elicited by UV-inactivated viruses. The response was MOI-dependent and immunoblots confirmed a correlation between luciferase induction and pp72-IE1 expression. Monoclonal antibodies, immune sera and purified immunoglobulin preparations decreased virus-dependent luciferase induction dose-dependently, qualifying this approach as surrogate virus neutralization test. Besides the reduced hands-on time, this assay allows analysis of herpesvirus entry in semi-permissive and non-adherent cells, which were previously non-assessable but play significant roles in herpesvirus pathology.

## Introduction

Herpes simplex virus 1 (HSV-1) and human cytomegalovirus (HCMV) are members of the *Herpesviridae* family representing prototypical α-herpesviruses and β-herpesviruses, respectively. Both viruses are ubiquitously found with seroprevalence of 50 to 100%. Herpesviruses are complex, enveloped, double-stranded DNA viruses, which show the remarkable ability to establish lifelong latency in immunocompetent hosts. Sterile immunity is never reached. Therefore, the presence of virus-specific antibodies is not only indicative for former infection events but also for replication competent virus able to reactivate under stress-full or immunocompromising conditions. Both viruses are continuously underestimated due to their often unapparent and subclinical infection, but account for severe and even fatal infections especially in immunodeficient individuals but also in apparently immunocompetent patients [1]. Additionally, HCMV ranges among the most-frequent, non-heritable, congenital diseases with intrauterine transmission rates of 30–40% upon primary HCMV infection during pregnancy [2]. Although a range of prevalence rates have been reported, recent studies indicate an incidence of congenital HCMV infection of 0.5–2% [2]–[4]. Approximately 10% of HCMV-infected newborns exhibit a symptomatic infection, which is frequently associated with sensorineural hearing loss and other sequelae [5]. According to the centers for disease control and prevention (CDC) approx. 1 in 750 children is born or develops permanent disabilities caused by HCMV (http://www.cdc.gov/cmv/facts.htm). Interestingly, incidence of HCMV transmission and severity of the associated morbidities seem to be reduced during recurrent episodes compared to primary infections [6]–[8], indicating that the adaptive immune response is capable to mediate some protection to the foetus.

The factors determining whether or not herpesvirus infections lead to symptomatic complications are incompletely understood. Studies in mice indicate that antibodies do play an important role in precluding recurrent mouse cytomegalovirus (MCMV) infection [9].

Immunoglobulins (Ig) are B-cell-derived, highly specific molecules for binding molecular structures of pathogens. Antibodies are grouped into the five different subclasses IgA, IgD, IgE, IgG and IgM, each having specialized functions. Abs function upon pathogen binding by Fc-receptor-mediated opsonisation, recruitment, activation of immune cells (like NK-cells, macrophages and B-cells) and triggering of the complement cascade. A minor fraction of antibodies is able to blunt infections directly by blocking essential mechanisms of attachment, entry or uncoating of intracellular pathogens like viruses [10]. These antibodies with direct antiviral capacity are referred to as neutralizing antibodies (nAbs).

Although the protection against herpesviruses and the control of reactivation has been attributed to T-cells, especially cytotoxic CD8^+^-T-cells and to a lesser extend CD4^+^ helper T-cells, it has now become increasingly evident that antibodies are important for immune control of cytomegaloviruses. It has been shown that the therapeutic administration of highly concentrated intravenous IgG (IVIG) preparations reduces the likelihood of congenital HCMV disease and thereby protects the foetus [11].

HSV-1 and HCMV are intermittently cytopathic viruses [12] with a delayed kinetic of nAb response after primary infection. For both viruses, the overall amount of antibodies measured by ELISA is not directly predictive for the amount of neutralizing antibodies in a given individual. In detail, nAbs against HCMV appear first approximately 13 weeks post primary infection and are continuously measured during reactivation [13], [14]. However, the inhibitory function of nAbs, particularly in HCMV infections differ in their blocking efficiency among different susceptible cell types [15] due to different neutralizeable protein complexes involved in virus entry [16]. Hence, assessment of the biological activity of neutralizing antibodies makes it necessary to perform time-consuming *in vitro* neutralization assays using different cell types.

The prerequisite for all HCMV and HSV-1 neutralization assays described so far are adherent and confluent cell layers of highly permissive cell types and either virus specific mAbs for staining of infected cells or direct counting of virus plaques [3], [13], [17]–[19]. *In vivo*, the route of entry into susceptible cells, and therefore the neutralizing activity of tested serum, is variable for HSV-1 and HCMV between different cell types [15], [20], [21]. Unfortunately, many cell types relevant for viral replication, transmission and pathogenesis are not suitable for testing using standard neutralization methods, so that the neutralizing capacity of antibodies can not be appropriately assessed. Furthermore, during analysis of borderline-concentrated HCMV-reactive sera in classical neutralization tests, like conventional micro-neutralization assays, high concentrations of IgG and serum proteins can cause an unspecific background stain of the cell monolayer (Reinhard H., unpublished observation). The routinely used methodology to test the neutralizing capacity of antibodies against herpesviruses relies on manual titration of virus and antibody dilutions, microscopic inspection and individual plaque counting. This procedure is time-consuming, laborious, error-prone and expensive. It does not allow high throughput approaches to routinely test large patient cohorts and thus remained a test principle for specialized laboratories.

Facilitating a promoter/enhancer element which is transactivated upon active herpesvirus infection, leading to expression of a reporter gene, we established a cheap, fast and easy new test principle, which allows the analysis of the neutralizing capacity of antibody preparations against herpesviruses in high-throughput approaches. The novel assay is not cell type restricted and generally applicable to a variety of cell types, even to non-adhering cells. Moreover, there is no need for visualizing infected cells by virus-specific antibodies and no need for manual plaque counting, opening new avenues to determine neutralizing antibodies in large patient cohorts and for large-scale screening approaches to identify therapeutic antibodies or pharmacologic inhibitors preventing herpesvirus entry.

## Results

### pTA vectors contain a herpesvirus responsive element

**Figure 1:**
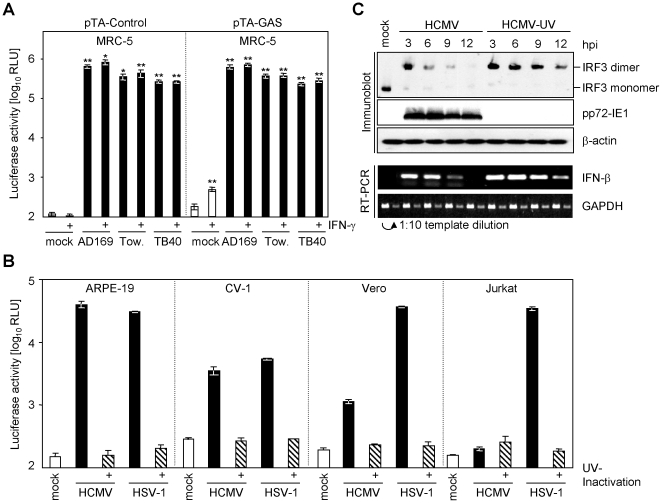
Herpesviruses transactivate the pTA-luc plasmid series. (**A**) Human MRC-5 cells, transiently transfected with the pTA-Control plasmid (left panel) or pTA-GAS (right panel), were split into aliquots and seeded. Cells were infected with 1 PFU/cell HCMV-AD169, HCMV-Towne, HCMV-TB40/E (black bars) or left uninfected (white bars). After 24 h the cells were treated for additional 5 h with 500 U/ml human IFN-γ. Cells were lysed and luciferase activity was measured. The experiment was performed in triplicate and the arithmetic mean with the standard deviation is shown. The significance was tested with the Student's t-test: * p<0.05 and ** p<0.01. (**B**) ARPE-19, CV-1, Vero and Jurkat cells were transfected with 2.5 µg pTA-Control plasmid using the Lonza nucleotransfection system. Cells were split so that parallel measurements and infections originated from the same transfection. 20 h post transfection cells were left uninfected (mock; open bars) infected with 3 PFU/cell HCMV-AD169, HSV-1 F strain (black bars) or UV-inactivated virus (hatched bar) for additional 20 h. Cells were lysed and luciferase activity was measured. (**C**) MRC-5 cells were infected with 3 PFU/cell HCMV-AD169 or UV-inactivated HCMV-AD169. After the indicated time cells were lysed and total RNA or protein was prepared. Protein lysates were subjected to native sodium-deoxycholate-PAGE for the analysis of IRF-3 dimerization or to SDS-PAGE for the analysis of pp72-IE1 and β-actin protein amount. Proteins were transferred to filters and probed with the indicated antibodies. IFN-β and GAPDH mRNA was assessed by semi-quantitative RT-PCR from total RNA as. For GAPDH two log_10_ dilutions were used as template to confirm measurement in the linear amplification range.

We observed a strong luciferase induction in cells transiently transfected with commercially available interferon-reporter constructs (pTA-GAS) upon infection with HCMV. Unexpectedly, similar luciferase expression was obtained from the corresponding control plasmid lacking the IFN-inducible promoter elements (pTA-Control) (Fig. 1a). In human MRC-5 lung fibroblasts both vector constructs (pTA-Control and pTA-GAS) were activated upon infection with three different HCMV strains, i.e. HCMV-AD169, HCMV-Towne and the endotheliotropic strain HCMV-TB40/E (Fig. 1a), suggesting that the vector backbone serendipitously contains a cytomegalovirus-responsive element. The virus-mediated activation resulted in a highly significant 1250- to 7000-fold luciferase induction (p = 0.0051 upon HCMV-AD169 infection of pTA-Control transfected cells). As expected, the IFN-γ-reporter-construct (pTA-GAS), but not the corresponding control plasmid (pTA-Control), was significantly induced upon treatment with IFN-γ (500 U/ml for 5 h) in mock-infected cells (Fig. 1a). The virus-specific response clearly exceeded the activation observed upon IFN-γ treatment (>1250-fold activation compared to ∼2.7-fold activation). Nevertheless, we used the control plasmid (pTA-Control), which is devoid of any additional promoter/enhancer elements, to preclude composite responses. The HCMV-specific response was in none of the cases significantly further increased upon treatment with IFN-γ (all t-test results >0.15) (Fig. 1a), which might be due to the well-described HCMV-encoded inhibition of Jak-STAT signalling [22], [23] or due to the fact that the virus-induced activation already reached saturating responses.

### The herpesvirus responsive element responds to different herpesviruses in different cellular contexts

In addition to responding to HCMV, increased luciferase expression upon infection of NIH3T3 cells with mouse cytomegalovirus (MCMV) was evident in a virus dose-dependent manner with both constructs (with or without an IFN-inducible promoter element), while only baseline luciferase expression was seen in mock infected cells (data not shown). Furthermore, HSV-1, an α-herpesvirus, induced luciferase activity in infected cells carrying a reporter constructs (Fig. 1b). Thus, this system was not limited to assessment of HCMV infection but responds to different types of herpesviruses. Furthermore, different cells of widely different origins mounted a significant signal upon infection (Fig. 1b). HSV-1, but not HCMV, induces luciferase expression in human Jurkat T-cells (Fig. 1b) confirming previous results, which revealed that HSV-1 is capable to enter T-cells [24]. In conclusion, this assay is adaptable to a wide range of target cells and it is not even limited to adherent cell lines but can be adapted to cells that grow in suspension.

### The herpesvirus responsive element is induced IFN-independently

Since it is well established that HCMV infection induces type I IFN (IFN-α/β) [25], we wondered whether the observed activation is the consequence of type I IFN induction. Especially the activation of the transcription factor IRF-3 is both hallmark and prerequisite for type I IFN induction. HCMV induce the formation of IRF-3 homodimers and the induction of IFN-β transcription (Fig. 1c). UV-inactivated HCMV induced an even more prolonged and increased IRF-3 activation and IFN-β transcription (Fig. 1c), likely due to the absent expression of virus-encoded inhibitors of IFN induction. We therefore tested whether the activation is also induced by UV-inactivated HCMV virions. As shown in Fig. 1b, UV-inactivated viruses did not activate the reporter construct. Thus, IFN-β and downstream signalling events can be excluded as potential activators because both, replication competent and UV-inactivated virus, induce IFN-β (Fig. 1c) but only the former induce increased luciferase expression.

Both HCMV and HSV-1 activated the reporter construct to induce luciferase in CV-1, Vero and ARPE-19 cells indicating that the activation is not cell type specific, although the expression of luciferase induced by HCMV in CV-1 is clearly weaker compared to ARPE-19 cells (Fig. 1b). Induction of luciferase by HCMV in Vero and CV-1 cells indicates that the assay principle is applicable to semi-permissive cells, because HCMV does not replicate in these cells. Since Vero cells harbour a deletion of the genes encoding for type I IFNs [26], the results obtained with Vero cells rule out an involvement of type I IFN genetically, consistent with the conclusion drawn above.

### Episomal vectors harbouring the promoter/enhancer element confer specific herpesvirus responsiveness

**Figure 2:**
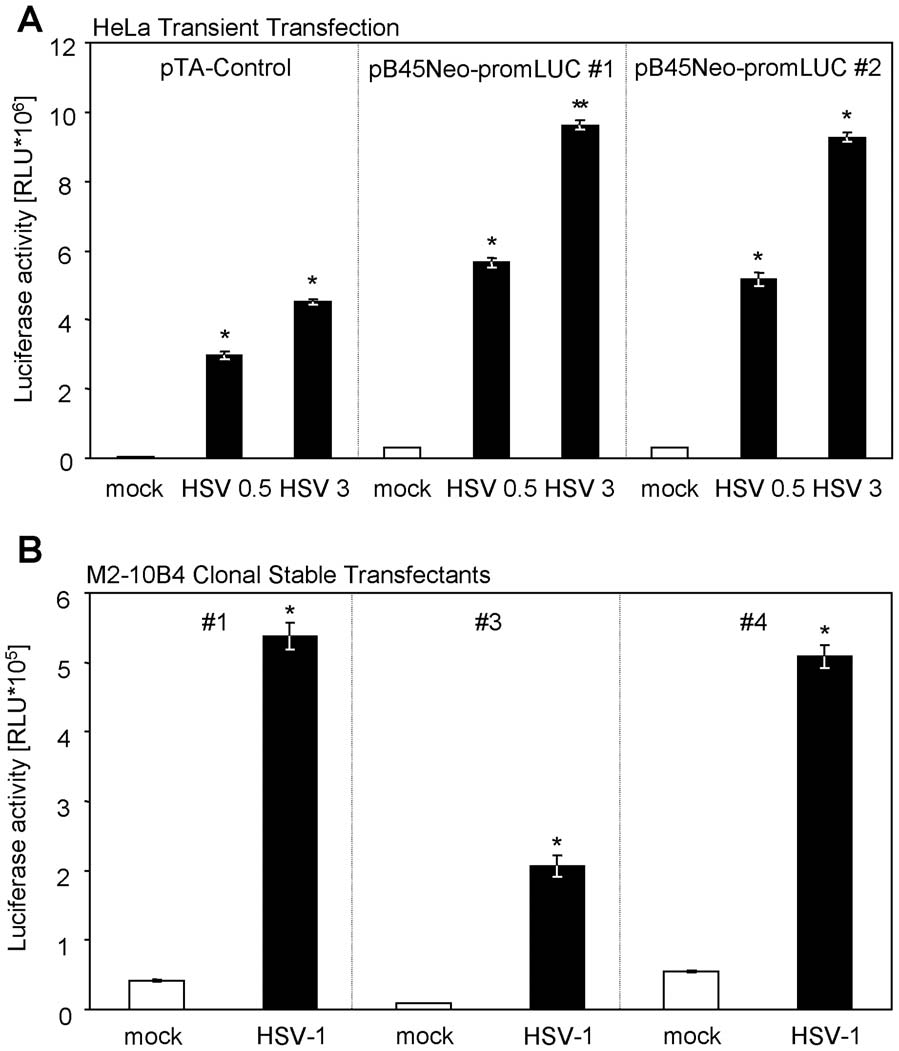
The episomal vector pB45Neo-promLuc confers herpesvirus responsiveness. (**A**) HeLa cells were transiently transfected with the pB45Neo-promLUC construct by nucleofection. Cells were infected with HSV-1 F strain (0.5 or 3 PFU/cell) or left uninfected. Cells were lysed and luciferase activity was determined. (**B**) G418-selected stable M2-10B4 cell clones (#1, #3 and #4) were infected with 5 PFU/cell HSV-1 F strain for 16 hours. Cells were lysed and luciferase activity was determined.

To allow establishment of stable cell lines harbouring the herpesvirus-responsive element, we sub-cloned the *luciferase* gene together with the corresponding promoter from the pTA-Control vector into a bovine papillomavirus-derived episomal vector (pB45Neo) [27]. Upon transient transfection of this vector (pB45Neo-promLUC) into HeLa cells, infection with HSV-1 induced luciferase expression (Fig. 2a). Clonal M2-10B4 cell lines harbouring pB45Neo-promLUC (selected by G418/geneticin) also responded to HSV-1 infection with robust luciferase induction (Fig. 2b).

**Figure 3:**
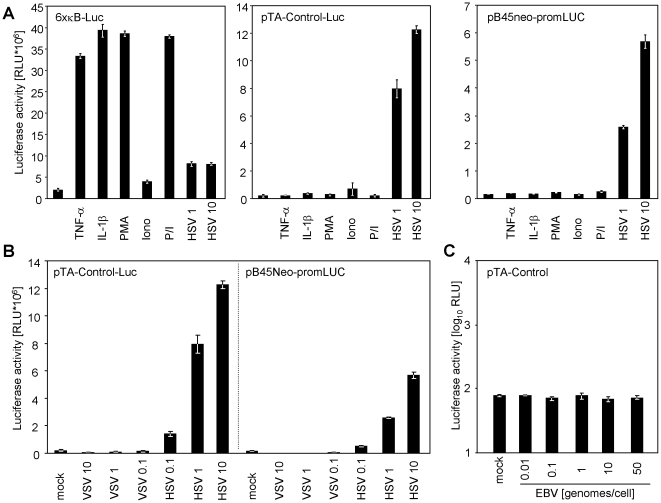
Specificity of responsiveness of the pTA-Control and the pB45Neo-promLUC construct. (**A**) HeLa cells were transiently transfected with 2 µg of the ‘6× κB-Luc’ NF-κB-reporter construct, the pTA-Control or the pB45Neo-promLUC. 16 h later cells were treated with 20 ng/ml tumour necrosis factor (TNF)-α, 5 ng/ml interleukin (IL)-1β, 0.1 µg/ml phorbol-12-myristate-13-acetate (PMA) or 1 µg/ml ionomycin (Iono) or both (PMA/Iono) for 4.5 h. A 16 h infection with 10 or 1 PFU/cell HSV-1 F strain (HSV 1 and HSV 10, respectively) served as control for inducibility by herpes viruses. (**B**) HeLa cells were transiently transfected with the pTA-Control or the pB45Neo-promLUC construct and infected with 0.1, 1 or 10 TCID50 vesicular stomatitis virus (VSV) and 0.1, 1 or 10 PFU/cell HSV-1 F strain, respectively. 15 h later cells were lysed and luciferase activity was measured. (**C**) 10^6^ RPMI8866 cells were transfected with 2 µg of the pTA-Control construct and infected over night with the indicated EBV genome copies/cell. Cells were lysed and luciferase activity was determined.

To analyze if the observed response is NF-κB-, PKC- or Ca^2+^-flux-dependent, we treated cells with either tumour necrosis factor (TNF)-α, interleukin (IL)-1β, phorbol-12-myristate-13-acetate (PMA) or ionomycin (Iono) and compared the responses to induction elicited by HSV-1 infection (Fig. 3a). To document the biologic activity of the used compounds, cells transiently transfected with the NF-κB-responsive “6× κB-Luc” reporter construct were conditioned in parallel. TNF-α, IL-1β and PMA/Iono induced luciferase expression from the NF-κB reporter but none substantially induced luciferase from either the pTA-Control or the pB45Neo-promLUC construct (Fig. 3a), indicating that HSV-1 infection induces luciferase by an alternative signalling mechanism.

Furthermore, no luciferase induction was observed upon infection with adenovirus (data not shown), the rhabdovirus vesicular stomatitis virus (VSV) (Fig. 3b) or the γ-herpesvirus Epstein-Barr-virus (Fig. 3c).

### HCMV and HSV-1 induce luciferase expression with differential kinetics

**Figure 4:**
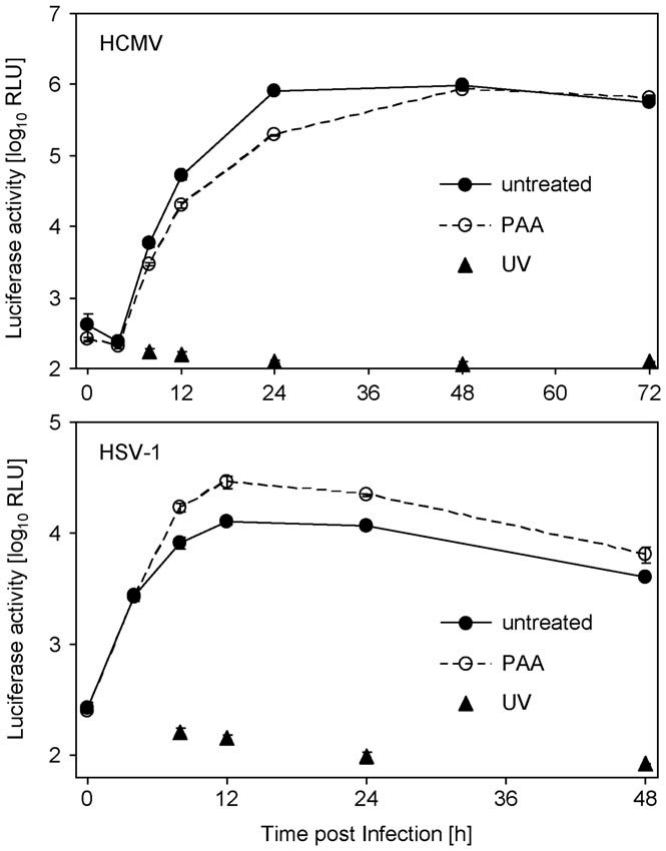
Time course of luciferase induction upon infection with HSV-1 and HCMV. MRC-5 cells were transfected with the pTA-Control plasmid by nucleofection and subsequently infected with HCMV-HB5 or HSV-1 F strain (4 PFU/cell). PAA was added at the time point of infection. Cells were lysed at indicated time points post infection and luciferase activity was measured.

We measured luciferase induction at different time points post infection with HSV-1 and HCMV. HCMV induced luciferase starting 8 h post infection, whereas HSV-1 induced luciferase already 4 h post infection (Fig. 4). Interestingly, we observed differences concerning the luciferase induction in the presence of phosphonoacetic acid (PAA), an inhibitor of herpesviral genome replication and accompanying *late* gene expression: HSV-1 induced increased amounts (∼2-fold 24 h post infection) of luciferase upon PAA treatment, whereas the HCMV-dependent response was diminished (∼4-fold 24 h post infection) upon PAA treatment (Fig. 4). We concluded that the transactivation is mediated by an *early/late* gene product in the case of HCMV but by an *immediate early* or strict *early* gene product in the case of HSV-1 (which is increasingly expressed in the absence of the feed-back inhibition by later gene classes – a regulative circuit which has been described previously [28]). The presented data indicate that the test principle is applicable during the entire viral life cycle and can be measured as early as 4 h and 8 h post infection with HSV-1 and HCMV, respectively.

### The reporter gene promoter in the pTA vector is transactivated

**Figure 5:**
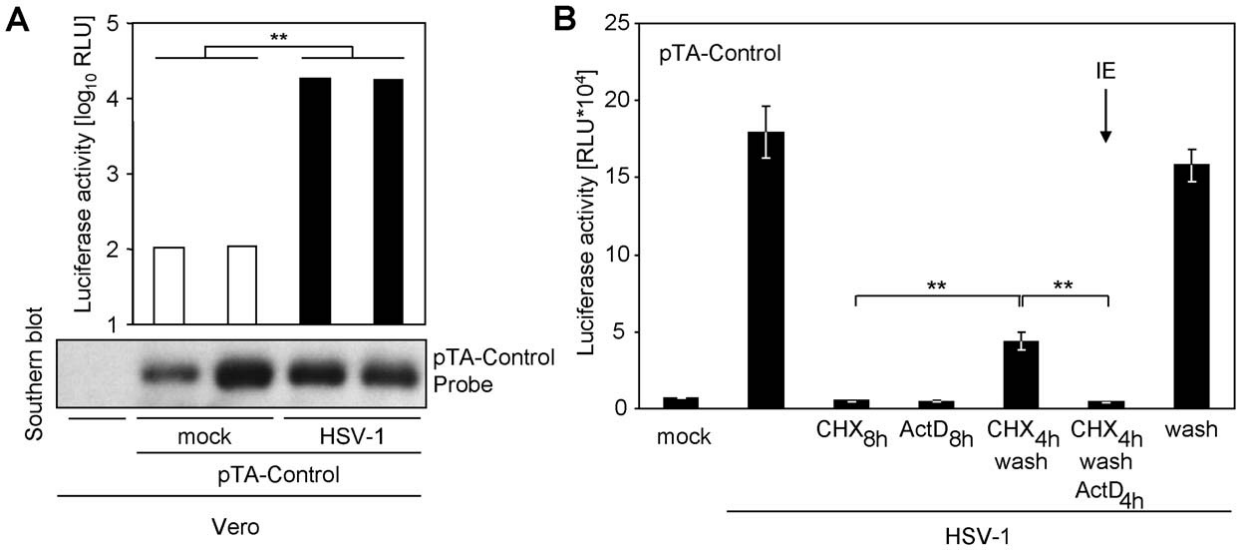
HSV-1 induces a bona fide transactivaion of the pTA-Control promoter. (**A**) Vero cells were transfected with the pTA-Control plasmid. The cells were split and infected with HSV-1 F strain. 24 h p.i. the expressed luciferase activity was measured. The cells were lysed and DNA was extracted in parallel, separated on an agarose gel and used for a Southern blot. The membrane was probed with DIG labelled (DIG high prime, Roche) pTA-Control plasmid. (**B**) CV-1 cells were transfected with 700 ng of the pTA-Control vector. Cells were infected with 10 PFU/cell HSV-1 F strain. Cells were incubated for 4 h with cycloheximide (CHX), which was replaced by 5 µg/ml actinomycin D (ActD) 4 h p. i. for selective immediate early protein expression conditions. Absence of ActD following washing (fifth bar f. l.) documents reversibility of CHX and continuous CHX and ActD treatment served as control for drug efficacy. Cells were lysed and luciferase activity was determined.

In general, two explanations for the increased luciferase expression are conceivable: (i) a virus-induced increase in plasmid number per cell or (ii) an increase in reporter gene expression per plasmid. The infection could significantly increase the number of plasmids per cell by either activation of the otherwise bacteria-specific origin of replication or by significantly increasing transfection efficiency *a posteriori*. We therefore tested by Southern blotting if transfected cells, which have been additionally infected, harbour more plasmid DNA than uninfected cells after transient transfection. We did not observe an increase of cellular pTA-Control amount upon HSV-1 infection, but a robust increase in luciferase expression in the same cells (Fig. 5a). We concluded that herpesviruses induce an increase of luciferase expression per plasmid by *bona fide* transactivation of the otherwise silent minimal promoter upstream of the luciferase gene.

Sub-cloning of the *firefly luciferase* gene together with 178 nucleotides (using a vector intrinsic *Not*I restriction site) of the upstream minimal promoter element into other vectors transferred the inducibility upon infection, indicating that this region harbours the transactivatable genetic element (data not shown). The subcloned fragment of the pTA-Control plasmid harbours two genetic elements, which could potentially affect expression: a transcriptional pause site and the minimal promoter. To distinguish these, we further truncated the promoter to 38 nts in front of the transcription start side using an intrinsic *Nhe*I-site. This promoter retained the ability to respond to HSV-1 (see data with pB45Neo-promLUC). Interestingly, the minimal promoter itself uses a TATA-box derived from the HSV-1 *thymidine kinase* (*tk*) gene, which has been shown to be significantly induced in transfected cells by HSV super-infection [29], [30]. To test the requirement of viral gene expression for transactivation, we exploited a well-known schema. Conditioning of the cells with the reversible translation blocker cycloheximide (CHX) only allowed transcription of immediate early mRNAs. Upon replacement of CHX by the RNA-polymerase II inhibitor actinomycin D (ActD) immediate early mRNAs, which have been transcribed in presence of CHX, become selectively translated. The absence of luciferase expression under such a regime (Fig. 5b), in conjunction with the results obtained upon UV-irradiation of the virus, indicated that viral gene expression is essential for luciferase expression. We concluded that these reporter plasmids harbour an HSV-1-derived *tk*-gene promoter element which is transactivated upon presence of *de novo* expressed viral transactivator proteins.

### HCMV and HSV-1 induce reporter gene expression MOI-dependently

**Figure 6:**
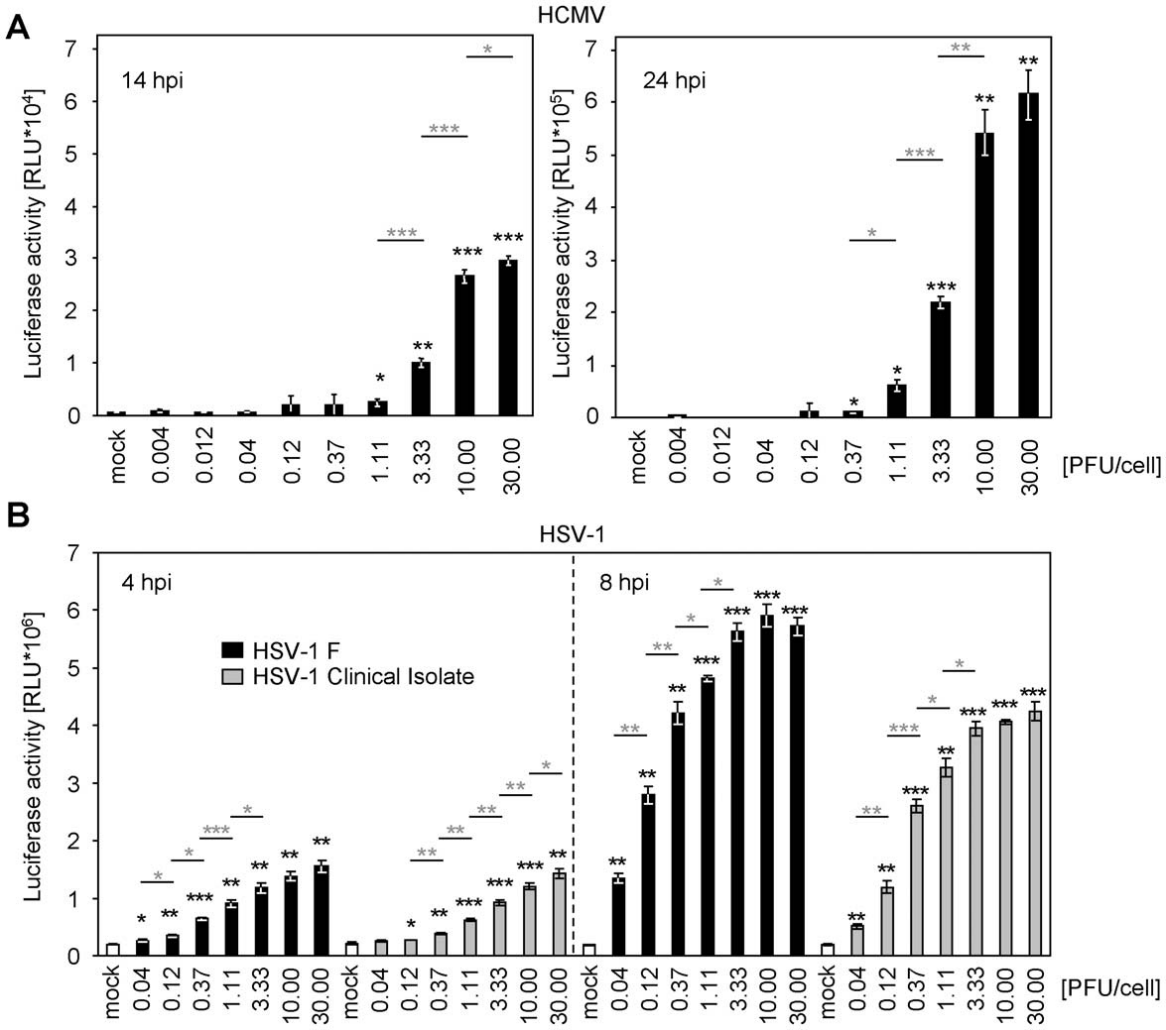
pTA-Control is transactivated upon herpesvirus infection in a virus dose-dependent manner. (**A**) WI-38 cells were transiently transfected (nucleofection) with the pTA-Control plasmid. Cells were infected for 14 h (left panel) or 24 h (right panel) with indicated PFU/cell HCMV-TB40/E. Cells were lysed and luciferase activity was measured. The arithmetic mean of triplicates ± standard deviation (SD) is shown. Statistical significance was tested by unpaired two-sided t-test with unequal variance compared to the mock (black asterisks) and the corresponding lower infectious dose (depicted in grey). * p<0.05; **p<0.01 and *** p<0.001. Please note the different scale between left (*10^4^) and right (*10^5^) panel. (**B**) As in (**A**) but HeLa cells were nucleofected with pB45Neo-promLUC and infected with the indicated infectious dose of HSV-1 F strain (black bars) or a low-passage clinical HSV-1 isolate (grey bars). Arithmetic mean of triplicates ± SD is shown. p-values as in (A).

To evaluate the potential value of this luciferase-based transactivation assay for the quantification of virus entry, we used serial dilutions of HCMV and HSV-1 over a wide range of infectious doses and analyzed the induced luciferase. Infection resulted in a virus dose-dependent luciferase expression spanning at least 2 orders of magnitude at early time points post infection (Fig. 6) and 3–4 orders of magnitude at later time points (data not shown). As expected, the system started to became saturated upon infection with higher MOIs (Fig. 6). We measured the viral reporter gene induction at early time points (4 and 8 h post infection with HSV-1 and 14 and 24 h post infection with HCMV) and observed a clear dose-response correlation. The assay was applicable to HSV-1 F-strain and a clinical HSV-1 isolate, which has been only passaged once in cell culture before the usage in this experiment (Fig. 6b – grey bars).

### Promoter transactivation can be exploited to measure virus neutralization

**Figure 7 pone-0014532-g007:**
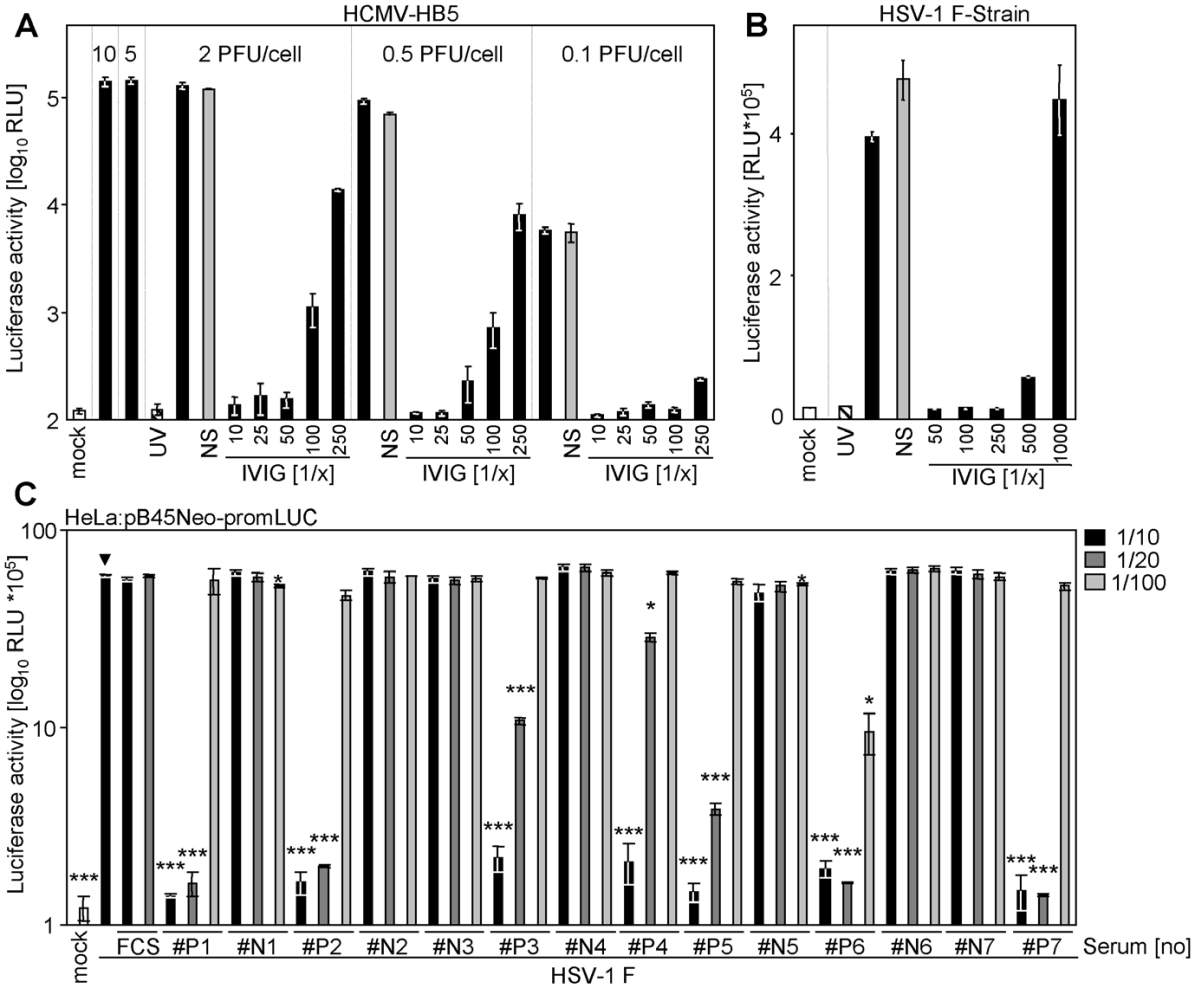
Virus-induced transactivation can be neutralized using IVIG. (**A**) MRC-5 cells were transfected with 2 µg pTA-Control plasmid using the Lonza transfection protocol and reagents. Cells were infected at different PFU/cell (10; 5; 2; 0.5 or 0.1 PFU/cell) of infectious HCMV-HB5 (black bars), UV-inactivated HCMV-HB5 (hatched bar) or left uninfected (white bar). Virus was incubated with indicated dilutions of the IVIG preparation Cytotect® for 1 h at 37°C before infection. As control the virus was incubated with the pooled sera of two seronegative donors (grey bar). (**B**) As in (**A**), but CV-1 cells were used and infected with HSV-1 strain F (2 PFU/cell). (**C**) 5 PFU/cell HSV-1 F strain was incubated for 90 min at 37°C with 1/10, 1/20 or 1/100 vol/vol dilutions of 14 human sera. #N1–#N7 HSV-1 seronegative donors and #P1–#P7 HSV-1 seropositive donors. pB45Neo-promLUC transfected HeLa cells were infected with virus-serum suspensions. 20 h post infection cells were lysed and luciferase activity was determined.

Since UV-inactivated HCMV and HSV-1 were not able to induce luciferase expression we concluded that the induction is due to a virus-dependent transactivation which requires viral gene expression. These findings prompted us to analyze whether virus neutralization of HCMV and HSV-1 by a commercially available IVIG preparation, Cytotect® (Biotest, Dreieich, Germany), is able to block transactivation. As shown in Fig. 7 the transactivation was again not detectable upon mock infection (white bars) or treatment with UV-inactivated HCMV (hatched bars). Infection with a replication competent HCMV or HSV-1 resulted in pronounced transactivation and luciferase expression. Pre-incubation with a non-immune serum pool (grey bars) did not decrease the virus-dependent transactivation, whereas pre-incubation with increasing concentrations of IVIG (black bars) neutralized transactivation dose-dependently, indicating that viral entry is a prerequisite for transactivation of the reporter construct-driven luciferase expression. Taken together, these results qualify this novel approach as a suitable surrogate assay for the determination of herpesvirus neutralization. Human sera of 7 HSV-1 seropositive individuals (#P1–#P7) and 7 seronegative individuals (#N1–#N7) documented a clear applicability for clinical sera: All 7 seropositive donors showed highly statistical significant neutralization of luciferase activity at 1/10 and 1/20 serum dilutions, whereas none of the seronegative donors showed signs of neutralization (Fig. 7c). One donor (#P6) showed significant neutralizing capabilities even at 1/100 serum dilutions.

**Figure 8 pone-0014532-g008:**
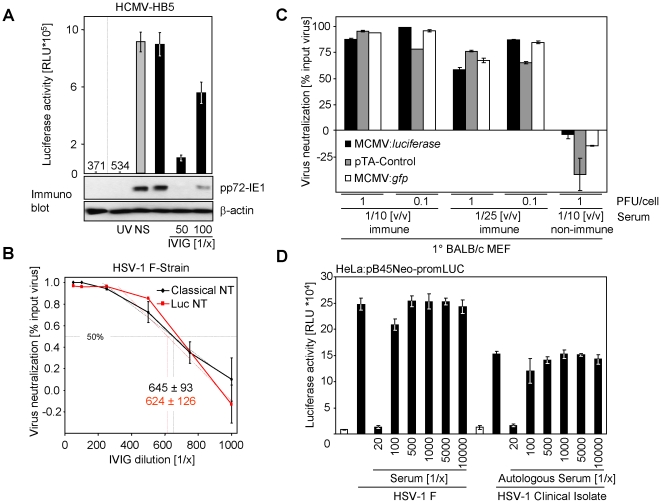
Comparison of new test principle with established methodology. (**A**) MRC-5 cells were transiently transfected (Lipofectamine) with pTA-Control plasmid. Cells were split and seeded before mock-infection or infection with UV-inactivated HCMV-HB5 or HCMV-HB5. Virus had been incubated with a 1/50 dilution of negative human serum (NS; grey bar), no serum or with the indicated dilutions of the IVIG preparation Cytotect® (black bars) for 1 h at 37°C. 20 h p. i. cells were lysed and luciferase activity was measured. An aliquot of the same lysates was separated by sodium-dodecylsulfate-PAGE. The proteins were transferred on filters and probed with pp72-IE1 and β-actin specific antibodies. (**B**) Comparison of the newly established luciferase-based neutralization test with a classical neutralization test. For the classical neutralization test, serial dilutions of Cytotect® were incubated for 1.5 h with 2 PFU/cell of the HSV-1 strain F prior to infection of CV-1 cells. 36 h p.i. plaques were counted. The mean of quadruplet values ± SD is shown. For the luciferase-based NT CV-1 cells were transiently transfected (Lipofectamine 2000 CD) with pTA-control plasmid, split and seeded. 2 PFU/cell of the HSV-1 strain F were incubated for 1.5 h with serially diluted Cytotect® before infecting CV-1 cells. 20 h p. i. the cells were lysed and luciferase activity was measured. The neutralization titer is determined as serum dilution able to mediate 50% virus neutralization. (**C**) Primary embryonic mouse fibroblasts were transfected by nucleofection with pTA-Control plasmid or left untransfected. wt-MCMV Smith strain, MCMV:*gfp* or Δm157-MCMV:*luciferase* were incubated for 90 min at 37°C with 1/10 (v/v) or 1/25 (v/v) dilutions of mouse (BALB/c) MCMV-immune serum or the control non-immune serum. Cells were infected with these suspensions. 16 h later cells were lysed for the determination of luciferase activity in the case of untransfected cells infected with Δm157-MCMV:*luciferase* and the pTA-Control transfected cells infected with wt-MCMV. GFP-positive cells were determined by FACS for MCMV-*gfp*. The relative neutralization of the input virus is depicted. (**D**) 5 PFU/cell HSV-1 F strain or a low-passage clinical HSV-1 isolate were incubated for 90 min at 37°C with indicated dilutions of the autologous human serum derived from the individual the virus was isolated from. 16 h later cells were lysed and luciferase was determined.

To validate the new test principle, we compared the virus-dependent transactivation with the expression of the HCMV protein pp72-IE1 measured by immunoblot after antibody-mediated virus neutralization. pp72-IE1 is expressed with immediate early kinetics upon infection and is not part of the HCMV particle and therefore constitutes a marker for successful HCMV entry and initiation of gene expression. As shown in Fig. 8a, UV-treatment of virus precluded luciferase expression and *de novo* pp72-IE1 expression. Non-immune serum did not decrease the reporter gene expression, whereas pre-incubation with IVIG effectively neutralized viral entry and gene expression. A strong correlation between luciferase activity and pp72-IE1 protein amounts - detected in the same lysate - became evident. These results validate that the luciferase induction constitutes an easy and quantitative surrogate for viral entry and subsequent gene expression.

To correlate the results obtained with the newly established luciferase-based neutralization test with classical micro-neutralization tests, we determined the neutralizing titer of IVIG in both assay systems simultaneously. As shown in Fig. 8b, both test systems gave almost congruent dose-response curves finally resulting in comparable 50% virus neutralization titers. Such a consistency between our newly established assay and a classical neutralization test was also observed upon HCMV neutralization (data not shown).

Since MCMV also transactivates the promoter, we compared the neutralizing capacity of MCMV immune serum and non-immune serum derived from isogenic inbred mice. We observed clear neutralization by immune serum at 1/10 and 1/25 serum dilutions, but not with non-immune serum, for two infectious doses (1 PFU/cell and 0.1 PFU/cell). Additionally, we compared our assay approach with neutralization test principles comprising luciferase-expressing MCMV and GFP-expressing MCMV. All three assays gave comparable results (Fig. 8c).

Having demonstrated the applicability for clinical HSV-1 isolates (Fig. 6b), we tested neutralization capacity of an ‘autologous’ serum-virus-pair from the same donor. We observed dose-dependent virus neutralization of reporter gene transactivation. Such a test might be of particular advantage since isolate/strain-specific neutralizing antibodies have been documented for HCMV gN [31]. These antibodies might now become easily assessable.

### Physicochemical parameters of HCMV entry and neutralization

**Figure 9:**
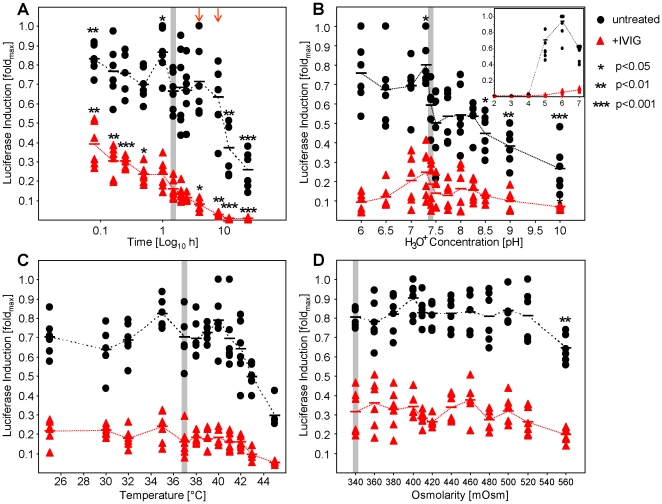
Neutralization test under different physicochemical parameters. MRC-5 cells were transiently transfected (nucleofection) with pTA-Control plasmid. 3 PFU/cell HCMV-TB40/E were incubated for 90 min at 37°C (or as indicated otherwise) with a 1/40 dilution of IVIG Cytotect® (red triangles) or antibody-free medium (black dots). After this incubation MRC-5 cells were infected. Luciferase induction was assessed one day post infection. Results are depicted as fold response compared to maximum response to allow comparison of the six independent experiments. (**A**) Effect of virus-antibody incubation time. (**B**) Impact of acidity (pH) on neutralization. Media was buffered to the indicated pH value by addition of HCl or NaOH, respectively. Arrows indicate time points (4 and 8 h) where intrinsic stability is not affected but neutralization is enhanced. An insert shows the neutralization at acidic conditions (pH 2–7). (**C**) Impact of temperature on neutralization during virus-antibody incubation. (**D**) Grading increase in osmolarity (by addition of NaCl; whereby 1 mM NaCl was calculated as 2 mOsm due to dissociation of ions) on neutralization. Standard conditions are shaded in grey. Statistically significant (two-tailed paired t-test) differences from these conditions are indicated by asterisks.

Facilitating our novel assay, we analyzed the impact of the parameters acidity (pH), osmolarity, temperature and incubation time on HCMV infectivity and virion neutralization. We chose an approximately half-maximal neutralizing IVIG concentration to analyze whether neutralization is increased or decreased. We incubated HCMV in presence or absence of IVIG prior to infection under the indicated conditions and subsequently infected cells maintaining the three remaining parameters as used under standard conditions (1.5 h incubation, 37°C, 340 mOsm, pH 7.4; indicated by grey shading). We focussed on a range of parameters, which we consider conceivable to occur under particular physiologic conditions. As shown in Fig. 9, acidity (pH 6–10), osmolarity (340–540 mOsm) and temperature (25–45°C) did not have a significant impact on virus neutralization. Increasing temperature reduced viral infectivity antibody-independently (Fig. 9c). Osmolarity had, at least under these experimental conditions, only limited impact on HCMV infection and neutralization (Fig. 9d). The pH seems to affect infectivity irrespective of antibodies and basic conditions (pH>8.5) seem to be more detrimental for HCMV than neutral and weak acidic conditions (Fig. 9b). Reducing the pH to 4 clearly damaged viral integrity, even in absence of antibodies, although statistically significant neutralization could still be observed. At pH of 3 or lower no viral transactivation was observed (see insert graphic of Fig. 9b).

Although the intrinsic HCMV stability at 37°C is limited (Fig. 9a), the incubation time between virus and antibody significantly affects neutralization: Incubating the virus without antibodies for 8 hours, 4 hours, 30 min, 15 min or 10 min did not resulted in significant changes in infectivity compared to standard conditions, whereas in the presence of antibody shorter incubation times (30, 15 and 10 min) decreased neutralization and prolonged incubation (to 4 or 8 hours, indicated by arrows) increased neutralization, respectively. We therefore suggest prolonging the incubation time to 4 or 8 hours (but not longer), especially for sera with borderline concentrations of neutralizing antibodies.

### The novel approach allows assessment of virus neutralization in non-adherent and semi-permissive cells

**Figure 10:**
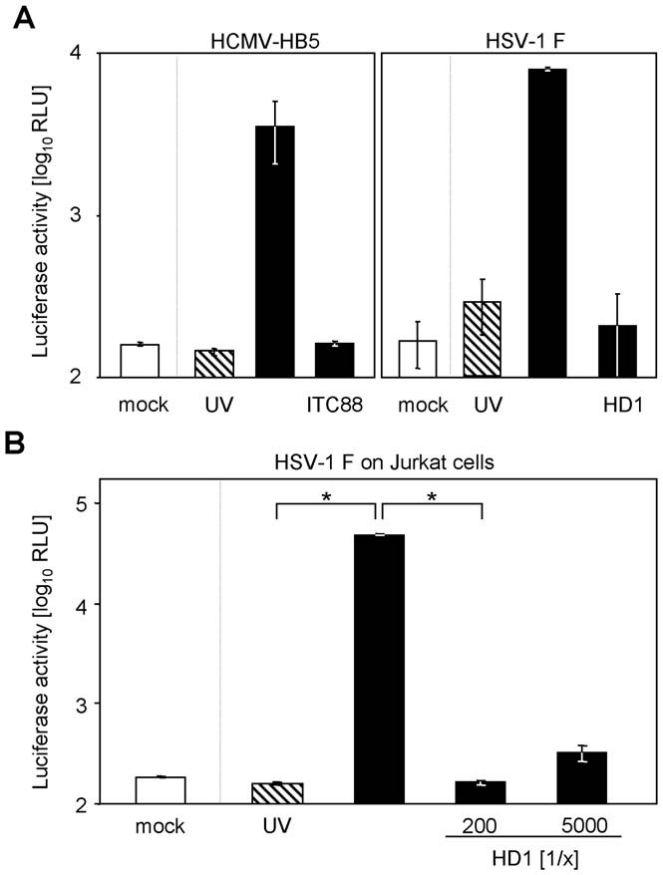
Viral transactivation can be neutralized by monoclonal antibodies in MRC-5 and Jurkat cells. (**A**) MRC-5 cells were transiently transfected (Lipofectamine 2000 CD) with the pTA-Control plasmid. Cells were infected with HCMV-HB5 (left panel) or HSV-1 strain F (right panel). Replication competent virus (black bars) was compared with UV-inactivated virus (hatched bars). HCMV was incubated for 1 h at 37°C before infection with 25 µg/ml of the HCMV gB-specific monoclonal antibody ITC88 and HSV-1 with the HSV-1 gD-specific monoclonal antibody HD1 before infection. Cells were lysed and luciferase activity was determined. (**B**) Jurkat cells were transfected with pTA-Control plasmid using the Lonza transfection reagents and protocol. Cells were infected with UV-inactivated HSV-1 strain F (hatched bar), replication competent HSV-1 (black bars) or left uninfected (white bar). Virus was incubated before infection for 1 h at 37°C with indicated dilutions of HSV gD monoclonal antibody HD1. ∼20 h p. i. cells were lysed and the expressed luciferase activity was measured.

The neutralization results obtained so far, using the IVIG preparation, prompted us to extend the assay application. We analyzed if well described neutralizing monoclonal antibodies against HCMV and HSV-1 are also able to block luciferase expression. The human monoclonal antibody ITC-88 (IgG1), directed against the AD-2 domain of the HCMV glycoprotein B (gB), a target of neutralizing antibodies during HCMV infection, has been shown to have neutralizing capacity [32]. Incubation with this nAb abrogated HCMV-induced luciferase expression (Fig. 10a). In a parallel set of experiments, neutralization by the HSV-1 gD specific monoclonal antibody HD-1 (IgG2a) [33] was assessed. Again the virus-induced luciferase expression was clearly neutralized. Altogether, these results confirm the usability of our novel luciferase-based reporter assay as neutralization test principle.

The reduced hands-on time and the objective read-out principle alone would already justify the use of the novel luciferase-based system to measure neutralization, but additionally, our results demonstrate, that the assay principle allows analysis of HSV-1 entry in non-adherent Jurkat cells. Classical neutralization assays are hardly applicable to suspension cells because neither plaque titration nor staining of infected cells can readily be performed. Since our test principle is neither restricted to adherent nor confluent cell layers which allow plaques formation upon infection, we surmised that our test principle would allow neutralization assays in cell types classically non-applicable. As proof-of-principle we tested if HSV-1 can be neutralized by the monoclonal antibody HD-1 in Jurkat cells. Significant transactivation was induced upon HSV-1 infection (p<0.013) but not by UV-inactivated HSV-1. The transactivation was abrogated upon treatment with the monoclonal antibody HD-1 (Fig. 10b). This result demonstrates that this new test system allows for the first time the quantitative analysis of neutralizing capacity of monoclonal antibodies, IVIG and patient sera in non-adherent and semi-permissive cells.

## Discussion

We observed significantly increased luciferase expression of cells transiently transfected with commercially available constructs initially designed to investigate Jak-STAT-mediated IFN signalling upon infection with different herpesviruses. This response was evident even with the otherwise silent control plasmid harbouring only a minimal promoter element.

The described assay principle facilitates herpesvirus-induced transactivation of a reporter construct to quantify virus entry and gene expression. The transactivation is measured by firefly luciferase expression after transfection of the reporter construct but other reporter genes could also be used. The inducibility was independent of the transfection protocols (data not shown). We show that the transactivation is not due to IFN induction but is a direct consequence of the entry of replication competent α- and β-herpesviruses (Fig. 1). The treatment with UV-inactivated virus was not sufficient to transactivate the promoter (Fig. 1), indicating that viral gene expression is necessary and that the transactivating principle is virus-encoded and not a cellular response to infection. Our data indicate that the promoter is transactivated by an *immediate early* or *early* gene product encoded by HSV-1 and by an *early*/*late* gene product of HCMV (Fig. 4).

The new assay allows quantitative and sensitive detection of herpesvirus infections over a large range of at least 2 orders of magnitude early after infection (and 4 orders of magnitude later post infection) and in a variety of different cell types - like MRC-5, Jurkat, Vero, CV-1, ARPE-19 (Fig. 1C) and NIH3T3 cells (data not shown) from different tissues (lung fibroblasts, T-cells, kidney cells and epithelial cells) and different species (*Homo sapiens*, *Cercopithecus aethiops* and *Mus musculus*). The reporter construct responded to different herpesviruses (HSV-1, HCMV and MCMV) and gave similar results when comparing different HCMV strains (HCMV-Towne, HCMV-HB5, HCMV-AD169 and HCMV-TB40/E) (Fig. 1a). The transactivation was blocked by IVIG preparations (Fig. 6–9), immune sera (Fig. 7c & 8d) and specific monoclonal antibodies (Fig. 10), but not by non-immune sera (Fig. 7 & 8), demonstrating that viral entry is a critical requirement for the transactivation. Therefore, the transactivation is a surrogate marker for successful viral entry and productive infection. Whether such a promoter element would also become transactivated upon reactivation from latency might constitute an interesting future issue.

Classical virus entry and neutralization assays are time-consuming and laborious, since a fixed number of plaque-forming virus particles is incubated with the blocking reagent (i.e. an antibody, serum or drug inhibitor) and than dilutions are transferred to permissive cells (usually highly permissive fibroblasts) to measure to which extent the treatment significantly reduces the number of plaques. Since the methods per definition operate with low countable virus/plaque numbers they are prone to handling errors and have large standard deviations making it necessary to measure large datasets to reach statistical significance. An obvious intrinsic disadvantage of such protocols is that they require virus plaque counting. Although automated plaque counting systems have been invented [17] they have not found their way into standard diagnostic laboratories. The plaque counting is not only time-consuming and laborious, requiring long hands-on time of trained personal, but is also very slow. Plaque formation of slow growing viruses like HCMV requires 6–8 days and even longer when analysing clinical HCMV isolates. Our method requires all together ∼24 h with a limited hands-on time and can even be reduced to 4–8 hours when stable transfectants are used.

Usual protocols comprise immunostaining procedures to specifically recognize virus protein producing cells before readable plaques are formed, but these protocols also take at least 3 days (from cell seeding to staining and plaque or foci counting) in the case of HCMV until results can be obtained and are therefore not as fast as our new approach and require even more hands-on time due to the staining procedure.

Future applications for this assay procedure will be to test antibody preparations and monoclonal antibodies for their antiviral efficiency against HSV-1 and HCMV on different cell types. The test can easily be used to analyze whether or not patients mount an effective neutralizing antibody response toward HSV-1 or HCMV. Additionally, the test could also be used to screen and analyze small molecular compounds for their ability to block HSV-1 and HCMV entry and fusion even in automated large scale screening approaches. Although not tested so far, it is tempting to speculate that other herpesviruses might share the capability to transactivate the responsive element and that it might thus be instrumental for the assessment of infection and neutralization of other human and non-human herpesviruses.

For HSV, transactivation-based entry and neutralization assays have been described [34]–[36], although in some reports composite responses have been measured, since transactivated promoters were combined with virus-driven reporter genes (*lacZ*) [34], [36]. To our knowledge, no such assay was available for HCMV until now.

In basic research such an assay system can be used for the assessment of the contribution of viral entry receptors and their ligands to HSV-1 or HCMV entry and fusion, e. g. UL128-131 [37]. Additionally, the ability of viral Fcγ-receptors encoded by HCMV [38]–[41] and HSV-1 [42], [43] to antagonize neutralizing antibodies can be studied. An obvious advantage of a cellular-intrinsic reporter system over a virus-driven one is the chance to work with unmodified viruses like clinical isolates, which in the case of HCMV clearly differ from cell-culture adapted viruses and the opportunity to directly compare sets of virus mutants with a common reporter system.

Since the identified herpesvirus responsive genetic element is silent in different cell types and cell lines and does not react on proinflammatory stimuli elicited by infection with UV-inactivated virus, even sophisticated experiments could be designed which would allow probing herpesvirus infection *in vivo*. It is tempting to speculate that transgenic animals like mice harbouring such a genetic element in front of reporter genes encoding for fluorescent proteins (i.e. eGFP or dsRed) or β-Gal in the *rosa26* locus would not only constitute a perfect sentinel mouse for animal facilities but also allow delineation of herpesvirus infected cells *in vivo*.

## Materials and Methods

### Cells and cytokines

Human MRC-5 lung fibroblasts passage 7–13 (ATCC CCL-171), human WI-38 fibroblasts (ATCC CCL-75), human HeLa (ATCC CCL-2), human retinal pigmented epithelium ARPE-19 (ATCC CRL-2302), African green monkey CV-1 (ATCC CCL-70), mouse M2-10B4 (kindly provided by Brendan Marshall [Medical College of Georgia, USA]) and Vero cells (ATCC CCL-81) were grown in Dulbecco's modified Eagle medium (D-MEM) supplemented with 10% (vol/vol) foetal bovine serum, streptomycin, penicillin and 2 mM glutamine. Jurkat cells (ATCC TIB-152) were maintained in RPMI media supplemented with 10% (vol/vol) foetal bovine serum, streptomycin, penicillin, 100 mM sodium pyruvate and 2 mM glutamine. Stable cell clones were selected using 250 µg/ml G418, GIBCO, Invitrogen. Human IFN-γ was purchased from PBL Biomedical Laboratories (New Jersey, USA). Cells were treated for 5 h with 500 U/ml. TNF-α and IL-1β were purchased from R&D Systems.

### Plasmids and transfection

The plasmids pTA-Control and pTA-GAS are part of the Mercury Pathway Profiling Luciferase Systems 5; catalogue number K2057-1 (lot# 2060828) (Clontech, Mountain View, USA). The plasmids were amplified in *E. coli* and DNA was prepared using a midi plasmid preparation kit (Qiagen, Hilden, Germany). The correct sequence of the minimal promoter pTA-Control was confirmed by DNA-sequencing (data not shown). The 6× κB-Luc NF- κB-reporter construct has been kindly provided by Klaus Schulze-Osthoff, Tübingen, Germany and has been described previously [44]. pB45Neo has been described [27]. For the generation of pB45Neo-promLuc, the intrinsic Mth-promoter, the intron and the poly-adenylation site sequences were excised by *Xba*I-*Bam*HI double-digest. The promoter and the *luciferase* gene were excised from pTA-Control using *Nhe*I (cohesive end to *Xba*I) and *Bam*HI and the insert was ligated into the cleaved pB45Neo.

Transfections were done transiently with 6 µl Lipofectamine 2000 CD reagent (Invitrogen, Carlsbad, USA) per 5×10^5^ cells or with the Lonza nucleofection electroporation method following manufacturer's instructions (Lonza, Cologne, Germany). MRC-5 cells were transfected using kit R (program X-001). WI-38 cells were transfected using Kit R (program V-001). Primary embryonic mouse fibroblasts were transfected using kit MEF2 (program A-23) and kit V was used for other cell types (programs as recommended by manufacturer). HeLa cells were transfected with Superfect, Qiagen, Hilden, Germany following manufacturer's instructions.

2.5 µg plasmid DNA was transfected per 5×10^5^ cells using the Lipofectamine transfection protocol. For nucleofection 2.5 µg per 10^6^ cells were used.

### Viruses and infection conditions

Purified stocks of HCMV strains AD169 [45], Towne (ATCC VR-977), the BAC-derived HB5 [46] and the BAC-derived endotheliotropic strain TB40/E [47] were used. Purified stocks of wt-MCMV strain Smith , MCMV:*gfp* and Δm157-MCMV:*luciferase* were used. MCMV:*gfp* has been described [48]. Δm157-MCMV:*luciferase* was kindly provided by Elke Bleifuss, HHU Düsseldorf, Germany. Purification was modified based on the method by [49] as described previously [50]. Infection was amplified by centrifugal enhancement at 800 g for 30 min at room temperature. The HSV-1 strain F was kindly provided by David Johnson (Portland, USA). Infections were done with a multiplicity of infection of 3 PFU/cell if not indicated otherwise. The low-passage clinical HSV-1 isolate was isolated from a healthy individual during characteristic reactivation (cold sore) and expanded once in cell culture. Crude stocks of VSV have been used and titrated by TCID50 determination on Vero cells.

### Neutralization, antibodies, serum

For neutralization experiments the virus was incubated with the indicated amount of monoclonal antibody, serum or IVIG for 1.5 h at 37°C in equal volumes of cell culture medium prior to infection of cells, which have been transfected before as described above. 20 h post infection luciferase activity was measured if not indicated otherwise. The IVIG preparation Cytotect® was a generous gift from Biotest (Dreieich, Germany) and concentrations were used as indicated. Of the human monoclonal anti-HCMV glycoprotein B (gB) antibody ITC88 (IgG1) ([32]) a concentration of 25 µg/ml was used in neutralization assays. The monoclonal HD-1 antibody (IgG2a), directed against HSV-1 glycoprotein D (gD), kindly provided by Gabriella Campadelli-Fiume (University of Bologna, Italy), was used at the indicated dilutions. Human sera were kindly provided by Ortwin Adams, HHU Düsseldorf, Germany. Usage of human sera for virus neutralization tests was approved by the ethical committee of the HHU Düsseldorf, Germany (#3414/2010). Collection of MCMV-specific latency sera was approved by the respective federal office (LANUV NRW; reference 50.05-240-61/06).

In the classical HSV-1 neutralization test, serial dilutions of IVIG were incubated for 1.5 h at 37°C in cell culture medium with an equal volume of virus preparation containing 2 PFU/cell. Afterwards CV-1 cells, grown to confluence in 48-well plates, were overlaid with the dilutions. After 36 h of incubation, virus-plaques were counted by microscopic inspection. The experiments were performed in quadruplets, shown is the arithmetic mean ± standard deviation. The calculated serum dilution inhibiting virus infectivity by 50% in comparison to untreated virus controls was indicated as the neutralizing-antibody titre. For a reasonable failure assessment we calculated the 50% neutralization rate based on a curve derived from the minimal or the maximal values of the quadruplet measurements of each antibody dilution, respectively. The curve leading to a higher deviation was used to calculate the depicted error of the 50% neutralization rate.

### Luciferase Assay

Luciferase activity was measured according to manufacturer's instructions (Roche, Mannheim, Germany) using a microplate luminometer (model LB 96V; Berthold).

### Immunoblotting

Immunoblotting was performed according to standard procedures. Briefly, luciferase assay lysates were prepared as described by the manufacturer (Roche, Mannheim, Germany). Lysates were subdivided, one aliquot was used to measure the luciferase activity and the other was used to determine pp72-IE1 and β-actin protein amount by immunoblotting. Proteins were separated by SDS-PAGE, transferred to nitrocellulose membranes and probed with anti-pp72-IE1 (Chemicon, Temecula, USA) and β-actin antibodies (Sigma-Aldrich, Taufkirchen, Germany) followed by a peroxidase-coupled goat anti-mouse secondary antibody (Dianova, Hamburg, Germany). Finally, proteins were visualized using the ECL-Plus chemiluminescence system (GE Healthcare, Munich, Germany).

### Southern blotting

Southern blotting was done following standard operation procedures with a nick translated DIG-labelled pTA-Control plasmid. Labelling was performed with the DIG-High Prime Kit (Roche, Mannheim, Germany) following manufacturers instructions.

### Analysis of IRF-3 dimerization and IFN-β induction

Analysis of IRF-3 dimerization by native sodium-deoxycholate-PAGE and RT-PCR for IFN-β and GAPDH were performed as described elsewhere [51]. Briefly, cells were lysed and total RNA was prepared using the RNeasy Mini kit together with the QIAShredder columns (Qiagen, Hilden, Germany). Semi-quantitative RT-PCR was performed using the OneStep RT-PCR kit (Qiagen, Hilden, Germany) using the following primers: 5′-ACCACAGTCCATGCCATCAC-3′ and 5′-TCCACCACCCTGTTGCTGTA-3′ for GAPDH. 5′-CTTTGCTCTGGCACAACAGGTAG-3′ and 5′-AGGATTTCCACTCTGACTATGGTC-′3 for IFN-β.
